# Management of transient ischemic attack in a low- and middle-income country: a critical analysis

**DOI:** 10.1055/s-0046-1818606

**Published:** 2026-04-07

**Authors:** Marcos Vinicius Sousa Varão, Guilherme Nobre Nogueira

**Affiliations:** 1 Universidade Federal do Ceará, Fortaleza CE, Brazil.; 2 Universidade Federal do Ceará, Hospital Universitário Walter Cantídio, Fortaleza CE, Brazil.

Dear Editor,

We would like to express out appreciation for the article “Transient Ischemic Attack in the Practice of Neurology in a Low- and Middle-Income Country”, by Moraes et al., 1 which addresses, in a relevant and timely manner, the clinical practices related to transient ischemic attack (TIA) within the Brazilian context. The study highlights important aspects of the healthcare scenario in low- and middle-income countries, shedding light on unique challenges, such as resource limitations, unequal access to diagnostic imaging, and difficulties in implementing standardized protocols. However, there are some topics that need further consideration.

The realities of a low-income country present several challenges, as many international studies tend to address practices in more privileged settings. Understanding the obstacles faced by Brazilian neurologists, such as restricted access to advanced imaging such as magnetic resonance imaging (MRI) and the scarcity of specialized TIA clinics, is of utmost importance to guide effective interventions. This perspective highlights the complex nature of the context and reinforces the need for tailored solutions aligned with local circumstances. Consequently, the study makes a valuable contribution to the development of more effective and inclusive health policies that can ultimately benefit vulnerable populations.

However, there is a topic about the treatment data that warrants clarification. The article presents an apparent statistical discrepancy by stating that “14 (56%) maintain treatment for up to 21 days [...] and 67 (56%), indefinitely.” This appears to be a probable typographical error, which is difficult to understand, as the absolute numbers (14 and 67) cannot correspond to the same percentage (56%). Clarification of this data point is important, as the duration of dual antiplatelet therapy (DAPT) is a cornerstone of management, with clear evidence supporting 21-day regimens for high-risk cases. 2

The research does not limit its scope and highlights that 80 (67%) of respondents reported the impact of the coronavirus disease 2019 (COVID-19) pandemic without TIA and stroke care. This broader perspective is commendable; however, the study does not present any information on what that impact was, treating the variable only as a demographic datum, and not as a variable of analysis.

Furthermore, the article successfully exposes the “critical inconsistencies” in the clinical practice. However, the non-response rate (65%) to crucial questions, such as the one regarding anticoagulation for cardioembolic TIA, suggests that conclusions about these specific practices should be interpreted with caution. Refining these aspects would enhance the value of the work as a scientific reference and strengthen its role in guiding future continuing education policies.

In summary, the article by Moraes et al. ^1^ offers an important and contextually-grounded contribution to the understanding of TIA management in low- and middle-income settings. By outlining the structural limitations and everyday clinical challenges, the authors provide valuable insight into the real-world practices of neurologists in Brazil. Nevertheless, addressing the stated topics would strengthen the manuscript and enhance its interpretability.
